# Predicting Response to Neoadjuvant Chemotherapy Using 18F FDG PET-CT in Patients with Locally Advanced Breast Cancer

**DOI:** 10.31557/APJCP.2020.21.1.93

**Published:** 2020

**Authors:** Narendra Hulikal, Sivanath Reddy Gajjala, Tekchand Kalawat, Silpa Kadiyala, Radhika Kottu

**Affiliations:** 1 *Department of Surgical Oncology, *; 2 *Department of Nuclear Medicine, *; 3 *Department of Radiology, *; 4 *Department of Pathology, Sri Venkateswara Institute of Medical Sciences, Tirupati, India. *

**Keywords:** Locally advanced breast cancer, neoadjuvant chemotherapy, clinical response

## Abstract

**Objective:**

The study was aimed to find the utility of 18F FDG PET CT in assessing response to neoadjuvant chemotherapy (NACT) in female patients with locally advanced breast cancer (LABC).

**Methods:**

All willing women with biopsy proven LABC, following clinical evaluation underwent baseline 18F FDG PET CT along with mammosonograpy and contrast enhanced computerized chest radiography (CECT). The response was assessed clinically before each cycle of chemotherapy using RECIST criteria. Those who were progressing clinically were offered alternate chemotherapy or radiation or surgery. Clinical responders were re-evaluated with 18F FDG PET CT, mammosonogram and CT chest before surgery. The pathological response as assed with residual cancer burden score was used as gold standard.

**Results:**

Of the 30 women eligible, 26 women underwent repeat evaluation and surgery. The mean age was 49 years, 16 women were postmenopausal and 15 tumors were receptor positive. On final histopathology 15 % had completer response and 46 % were non responders. Using a cut off value of 50% of the baseline SUV_max_, PET-CT had sensitivity, specificity, positive predictive value, negative predictive value and accuracy of 64%, 75%, 75%, 65%, and 69%, respectively in differentiating pathological responders from non-responders.

**Conclusion:**

18F FDG PET-CT predicted the response with greater accuracy than CT or clinical examination. Hence it can be used to identify non responders early in the course and alternate treatment can be offered to patients.

## Introduction

Neoadjuvant chemotherapy (NACT) has become the initial treatment of choice for majority of locally advanced breast cancer (LABC). The responders are then offered locoregional treatment comprising of surgery and radiation therapy (Tryfonidis et al., 2015). Depending on the receptor status hormonal treatment is later administered for a minimum of five years. In this sequence of treatment assessing the response to chemotherapy is an important step for optimal timing and achieving good therapeutic outcome for the individual patient. The tools available for assessing the response are mainly clinical examination at each cycle of chemotherapy, mammosonograpy, contrast enhanced computerized tomography (CECT) and magnetic resonance imaging (MRI). Due to many limitations with these anatomical methods of assessing response such as difficulty in differentiating between scarring and residual tumor newer techniques have gained popularity in recent times (Abraham et al., 1996; Helvie et al., 1996; Vinnicombe et al., 1996; Herrada et al., 1997). Two of the emerging techniques include 18 flouro fluorodeoxy glucose (18F FDG) positron emission tomography computerized tomography (PET-CT) scan and dynamic contrast enhanced MRI. Further good response to NACT being a surrogate marker of prognosis, helps in appropriate management of the patient if it can be assessed early in the course of treatment with accuracy (Minckwitz et al., 2008). In this regard 18F-FDG PET-CT can assess metabolic as well as morphological response as early as 2nd or 3rd cycle when done at baseline. Hence this study was planned to know the usefulness of 18F FDG PET-CT in assessing the response of tumor to NACT in patients with LABC.

## Materials and Methods

This was a prospective study of all willing, women with biopsy proven, unilateral, newly diagnosed LABC (defined as those belonging to stage III, AJCC/TNM, 7^th ^edition, 2010) receiving NACT over a period of 20 months in a tertiary care cancer hospital in southern India. Women with early or metastatic breast cancer, uncontrolled diabetes, pregnancy and recurrent or treated elsewhere were excluded from the study. The study protocol was approved by the institutional ethical committee and all tests were done free of cost through institutional funding. All eligible women underwent a baseline 18F FDG PET-CT as per the institutional protocol described elsewhere (Gajjala et al., 2018). Any women proven to be metastatic were excluded from the study. All women were evaluated with a baseline CECT and Mammogram. All the eligible women received standard chemotherapy regimen as per the protocol of the department of Medical Oncology. Due to financial constraints on part of the patient, no anti her 2 nu treatment was employed in any of the patients either pre or post-surgery. Before starting new cycle of chemotherapy, a clinical examination was done to assess the response. Those with obvious clinical response as perceived by a senior clinician continued to receive chemotherapy and were subjected for a repeat PET-CT at the end of 3rd or 4th cycle, whereas non-responders had a repeat scan at the completion of 2^nd^ or 3^rd^ cycle. For clinical and CT analysis maximum diameter of breast lesion in one plane was used. Lesions that decreased in size by 50% or more were classified as responders, and those lesions with less than 50% decrease in size were classified as non-responders. For PET, a SUV max reduction of more than 50% of baseline was used to differentiate responders from non-responders. Lesions with reductions in SUV_max _of 50% or more were classified as responders, and those lesions with less than 50% decrease in SUV_max_ were classified as non-responders. Any patients with disease progression were offered surgery if it was feasible. Any metastatic disease would have dictated exclusion. The surgery was done 3-4 weeks after the last chemotherapy cycle and once all blood parameters were normalized. Before surgery a symptom directed metastatic work up and mammography was done. All patients underwent modified radical mastectomy. The pathological response was assessed by histopathological analysis of operative specimen by a single pathologist. Based on final histopathology, residual cancer burden (RCB) was calculated using RCB calculator from www.mdanderson.org and the patients were classified into four RCB classes pathological complete responder (pCR), RCB- I, RCB – II, RCB-III. Patients in RCB – III were considered as non- pathological responders (non-pR) and the rest as pathological responders (pR). Following surgery patients received locoregional radiotherapy and further treatment as per standard guidelines.

*Statistical analysis*

Sensitivity, specificity, positive predictive value, negative predictive value, and accuracy were determined for clinical examination, CT and PET-CT in assessing treatment response using pathological response as the gold standard. The Mann Whitney U test was used to compare SUVs on PET-CT, size of tumor on clinical examination and CT between pCR and non-pCR group and between pR and non-pR. For all tests, an alpha error up to 5% (P< 0.05) was considered significant. Receiver operating curve (ROC) analyses was used to determine the optimal cut-off values of change in SUV that better discriminate pCR from non-pCR, and pR from non-pR patients.

## Results

During the study period a total of 30 patients were eligible, of which 26 patients completed NACT and underwent surgery. Mean age was 49 years, majority were postmenopausal (16 of 30) and 15 women had hormone receptor positive and 9 had triple negative tumors. The timing of reassessment PET-CT was after 2 cycles in 11, after 3 cycles in 9 and after 4 cycles in 6 patients. On histopathological examination 14 (54%) patients had pathological response and 12 (46%) had no response. Pathological complete response (pCR) was seen in 4 (15%) patients, RCB class I in none, II in 10 (39%) and III was present in 12 (46%) patients.

**Figure 1:**
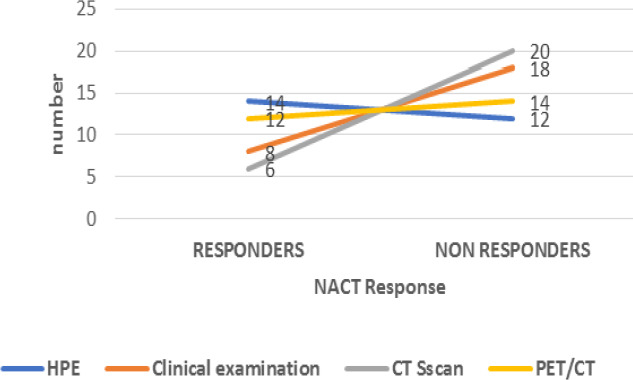
Response Assessment with Different Modalities

We compared clinical examination, CT and PET/CT in their ability to differentiate PCR from non-PCR and pR and non-pR (Figure 1). There was no significant difference in the mean baseline tumor size, mean follow up tumor size and mean change in tumor size on clinical examination and CT between pR and non-pR groups; pCR and non-pCR groups. The mean Suv_max_ at baseline PET-CT was not significantly different among pR and non-pR groups (P =0.8990) and among pCR and non-pCR groups (P=0.918). At interim PET-CT the mean Suv_max_ of the patients with pCR (1.12, SD- 1.29) was significantly lower than the non-pCR (10.43, SD- 9.25) (P = 0.01). Although there was decrease in mean SUV max, there was no significant difference among pR and non-pR (P= 0.193). The mean percentage change in the SUV_max_ of the primary tumor on the follow-up examination as compared to the baseline examination was 52.18% (SD- 42.9) among pR and 29.73%(SD-47.06) among non-pR (P=0.252). The mean percentage change in SUV max of primary tumor on follow up scan was significant in pCR (90.67, SD 11.76) than non –pCR (32.94, SD 43.56) patients (P=0.04) (Tables 1 to 3).

*Comparison of various modalities with HPE in evaluation of response to NACT*

**Figure 2:**
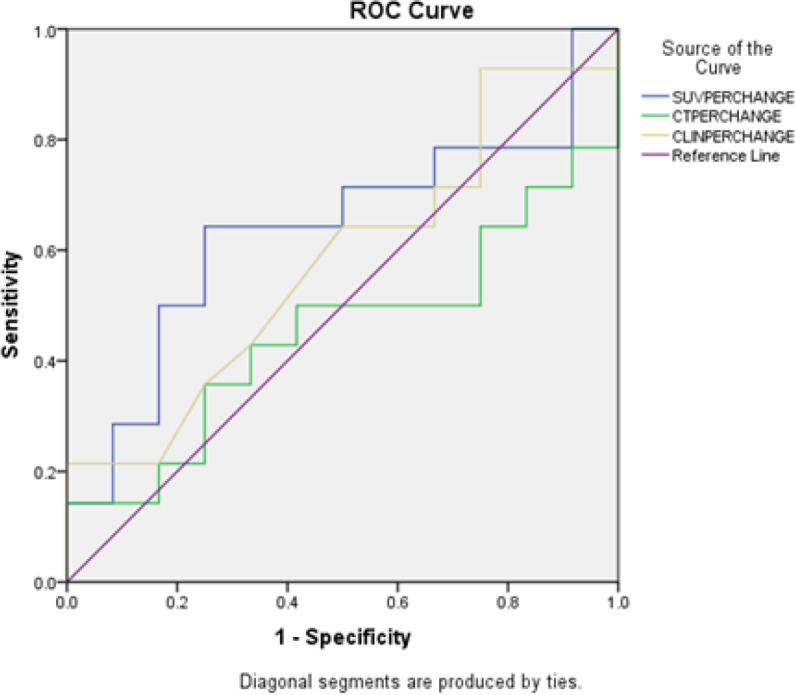
Receiver Operating Curve (ROC) Analyses for the Prediction of Pathological Response

**Figure 3:**
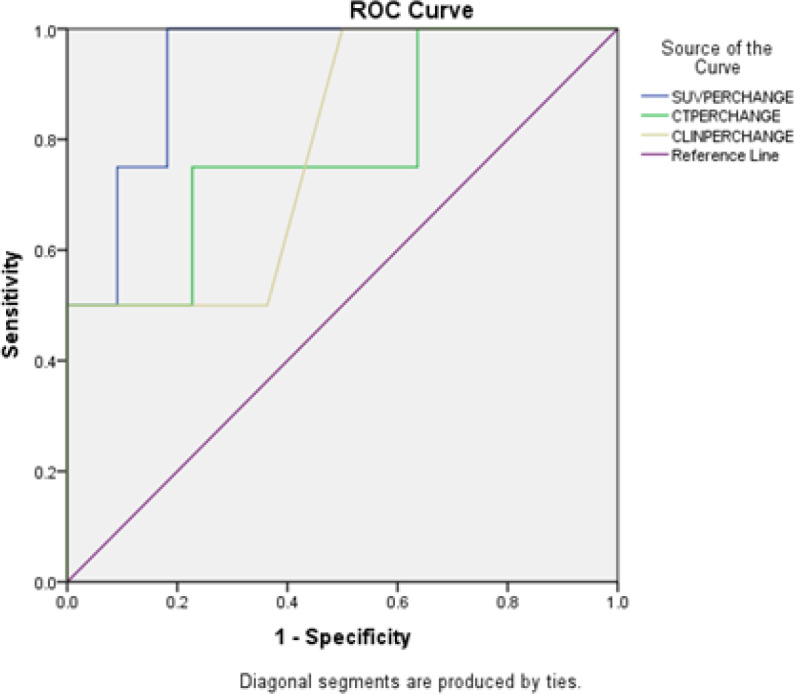
Receiver Operating Curve (ROC) Analyses for the Prediction of Complete Pathological Response

Using a cutoff value of 50% of the baseline SUV_max_, PET-CT had sensitivity, specificity, PPV, NPV and accuracy of 64%, 75%, 75%, 65%, and 69%, respectively in differentiating pR from non-pR. The above values for clinical examination and CT evaluation were 43%, 67%, 60%, 50%, 54% and 22%, 75%, 50%, 45%, 46% respectively (Table 4). The details of the comparison are given the Table 4. A receiver operating curve (ROC) analyses for the prediction of pR was done (Figure 2). From the curve the decrease in SUV max of 48.87% is optimal to discriminate between pR and non-pR with sensitivity and specificity of 63.4 % and 75% respectively. A ROC analysis for prediction of pCR was then done (Figure 3). From the curve the decrease in SUV max of 85.85% is optimal to discriminate between pCR and non-pCR with sensitivity and specificity of 75 % and 91% respectively.

**Table 1:** Clinical Tumor Size in Comparasion to Pathological Response at Baseline and on Follow up

Clinical examination time line	Pathological response group	Mean Tumor Size (cm)	Standard deviation	*p-value* (Mann-Whitney U test)
Baseline	pR	6.42	1.69	0.595
	Non-pR	6.92	1.93	
	pCR	6.75	2.22	0.811
	Non-pCR	6.64	1.76	
Follow up	pR	3.82	2.59	0.462
	Non-pR	4.33	1.23	
	pCR	2.25	2.87	0.15
	Non-pCR	4.39	1.77	
Change (%)	pR	42.67	35.31	0.494
	Non-pR	33.79	23.18	
	pCR	70	34.64	0.081
	Non-pCR	32.85	26.15	

**Table 2:** The Mean CT Tumor Size at Baseline vs at Follow up

CT scan	Group	Mean Tumor Size	Standard deviation	*p-value* (Mann-Whitney U test)
Baseline (cm)	pR	5.27	2.22	0.252
	Non-pR	6.25	2.51	
	pCR	6.00	2.95	0.811
	Non-pCR	5.67	2.32	
Follow up (cm)	pR	3.46	2.71	0.82
	Non-pR	3.36	0.90	
	pCR	2.10	2.60	0.283
	Non-pCR	3.60	1.90	
Change (%)	pR	36.95	36.25	0.705
	Non-pR	40.16	22.49	
	pCR	69.16	36.49	0.081
	Non-pCR	32.85	26.05	

**Table 3:** The Mean Suv_max _ at Baseline and Follow up PET-CT

PET-CT Evaluation	Group	Mean	Standard deviation	*P-value *(Mann-Whitney U test)
Baseline (Suv_max_ )	pR	16.12	8.36	0.899
	Non-pR	16.12	9.41	
	pCR	14.17	3.83	0.918
	Non-pCR	16.48	9.31	
Follow up (Suv_max_ )	pR	7.81	8.66	0.193
	Non-pR	10.39	9.91	
	pCR	1.12	1.29	0.01
	Non-pCR	10.43	9.25	
Change in SUV_max_ (%)	pR	52.18	42.9	0.252
	Non-pR	29.73	47.06	
	pPCR	90.67	11.76	0.04
	Non-pCR	32.94	43.56	

**Table 4:** Comparison of Various Modalities with HPE in Assessing NACT Response

		Pathological response		Prediction of response (%)				
		Responders	Non responders	Sn	Sp	PPV	NPV	Acc
Clinical response	Responders	5	3	38	75	62.5	50	54
	Non responders	9	9					
CT response	Responders	3	3	22	75	50	45	46
	Non responders	11	9					
PET/CT response	Responders	9	3	64	75	75	64	69
	Non responders	5	9					

Sn, Sensitivity; Sp, Specificity; PPV, positive predictive value; NPV, negative predictive value; Acc, Accuracy.

**Table 5:** Studies using PET or PET-CT for evaluating NACT response in breast cancer

Author, Year	n	AJCC Stage	Baseline PET n	1^st^ PET n	2^nd^ PET n	3^rd^ PET n	Tumor response	Comments
Schelling et al., 2000	22	LABC	22	14 (1 cycle)	20 (2 cycles)	7 (after completion)	MRD	Sn 100, Sp 85- At 1 cycle; Sn 83, Sp 94- At 2 cycles
Smith et al., 2000	30	T3 or LABC	30	28 (1 cycle)	19 (4 cycles)	21(8 cycles)	pCR micro/macro	Sn 90, Sp 74 - At 1 cycle
Kim et al., 2004	50	LABC	50	50 after completion			Non-pR pR pCR	Sn 85, Sp 83- after completion
Rousseau et al., 2006	64	II, III	64 (PET/CT)	64 (1cycle)	64 (2 cycles)	64 (3 cycles)	GRD, MRD	At 50 cutoff Sn 39, Sp 96-At 1 cycle Sn 69, Sp 89- At 2 cycles Sn 79, Sp 77- At 3 cycles
Li D et al., 2007	45	NA	45 (PET/CT)	45 (3 Cycles)	-	-	Apoptotic index	Sn 91, Sp 83- At 3 cycles
Kumar et al., 2008	23	II, III	23 (PET/CT)	23 (2 Cycles)	-	-	Non-pR, pR	Sn 93, Sp 75-At 2 cycles Accuracy 87
Andrade WP et al., 2013	40	II, III	40 (PET/CT)	40 (2 cycles)	40 (before surgery)	-	RCB protocol	At SUV 59.1 Sn 68, Sp 75
Present study	26	LABC	26 (PET-CT)	11 (2 cycles), 9 (3 cycles), 6 (4 cycles)	-	-	RCB protocol	At SUV 50 Sn 64, Sp 75, accuracy 69

n, number of patients; Sn, sensitivity (%); Sp, specificity(%); pR, pathological responder; pCR, pathological complete response; micro, microscopic; macro, macroscopic; GRD, gross residual disease; MRD, macroscopic residual disease; NA, not available.

## Discussion

It has been shown that early response after two or three cycles of chemotherapy can be a predictor of pathologic complete remission and may therefore serve as a predictor for long-term outcome (Minckwitz et al., 2008). Around 70% of patients demonstrate clinical response to NACT and only about 20% achieve pCR (Bonadonna et al., 1998; Hage et al., 2001; Fisher et al., 2002). In comparison with non-PR, patients with pCR or minimal residual disease have longer disease-free and overall survival rates (Feldman et al., 1986;Fisher et al., 2002). Therefore, methods that allow prediction of therapeutic effectiveness at an early time point could help to individualize treatment, switch over to non-cross resistant chemotherapeutic agents and to avoid potentially ineffective chemotherapies. PET or PET-CT can predict pathologic response to NACT early in the course (Schelling et al., 2000; Smith et al., 2000; Rousseau et al., 2006; Dose et al., 2009). Currently, apart from clinical evaluation, the anatomical imaging modalities such as mammography, ultrasonography, CECT and MRI are used to assess the response primarily by evaluating the change in size of the tumor. However, limitations of these include their limited accuracy and reproducibility in determining tumour size and the time lag between initiation of therapy and detectable tumour shrinkage (Abraham et al., 1996; Helvie et al., 1996; Vinnicombe et al., 1996; Herrada et al., 1997). Furthermore, in patients with residual masses after therapy, anatomical imaging does not distinguish viable tumour tissue from fibrotic scar tissue. As the change in tumour metabolism precedes the decrease in tumour size, FDG PET should allow visualization of tumour response at an earlier stage than with conventional imaging methods. Wahl et al., (1993) were among the first to show that serial FDG PET imaging allows differentiation of responders versus non-responders, by measuring changes in tumor FDG SUVs with treatment. Subsequent studies showed that pathologic response to NACT in LABC can be predicted accurately by FDG PET or PET-CT early in course (Schelling et al., 2000; Smith et al., 2000; Rousseau et al., 2006; Dose et al., 2009). Rousseau et al., (2006) reported on the efficacy of FDG PET-CT for evaluating early response to NACT in 64 patients with stage II and III breast cancer who underwent PET after the first, second, third, and sixth courses of chemotherapy. Using a 60% decrease in baseline SUV as their threshold for response, they found that FDG PET was 61% sensitive and 96% specific after a single cycle, and this increased to 89% sensitive and 95% specific after two cycles of therapy. Kumar et al., (2009) showed that at 50% reduction in SUV max, PET-CT had sensitivity, specificity and accuracy of 93%, 75% and 87% respectively for differentiating pathological responders from non-responders.

The time lag between chemotherapy and the morphologic response ranges anywhere between 4-6 weeks, which is too long. Furthermore, with the morphological imaging it is difficult to distinguish fibrosis with the viable tumor residue and the presence of scarring, edema and inflammatory reaction in the post chemotherapy setting can lead to misclassification of a chemo-sensitive tumor into a non-responder (Wolfgang and Weber, 2009). With the availability of newer chemotherapy agents, it becomes important to measure the efficacy of these chemotherapy regimens early in the course of treatment so that ineffective chemotherapies can avoided in non-responders and alternative drugs may be employed. In the present study we found that PET-CT was more accurate (87%) than clinical (39%) and CT (56%) for response evaluation. This can be explained by its capability to detect metabolic changes, which takes place much earlier than structural changes. A detailed review of the studies on PET or PET-CT in evaluating treatment response in breast cancer is described in Table 5. There is a large variation in sensitivity and specificity, ranging from 39% to 100% and 74% to 100%, respectively. The variation in sensitivity and specificity in various studies can be explained by different patient inclusion criteria (stage), different timings of PET, different cutoff SUVs, and different pathological criteria.

Our study suggests that the decrease in SUV max of 48.87% is optimal to discriminate between pR and non-pR with sensitivity and specificity of 63.4 % and 75%. Andrade et al., (2013) suggested the optimal threshold of decrease in SUVs of 59.1% to discriminate between pR and non-pR (or RCB-III) after the second cycle of chemotherapy, with positive predictive value of 50.0%, negative predictive value of 70.0% and accuracy of 86.3%. Martoni et al., (2010) studied 34 patients and the decrease in SUV with optimal negative predictive value to predict pathologic response was 50%. Kumar et al., (2009) also found a SUV cutoff of 50% of baseline with accuracy of 87%. Dose et al., (2009) published a multicenter trial with 104 patients where 81 were evaluated after the second cycle and change in SUVof 55% predicted pathologic response with a sensitivity of 69%, specificity of 63%, and negative predictive value of 89%.

The optimal timing for the interim PET remains unclear. For several teams, performing PET after the second course of NACT is a good compromise to evidence effects of chemotherapy and to still allow an early change of treatment in case of ineffectiveness (Schelling et al., 2000; Rousseau et al., 2006). Studies performed after the completion of chemotherapy have shown that while residual FDG uptake is predictive of residual disease, the absence of FDG uptake is not a reliable indicator of complete pathologic response (Bassa et al., 1996; Burcombe et al., 2002; Kim et al., 2004). This is especially true for axillary nodal disease, since the sensitivity for residual microscopic disease is low. Similarly, the cutoff value of baseline SUV max to differentiate responders from non-responders varied among different studies. Most of the studies it was in range of 40 to 60% (Schelling et al., 2000; Rousseau et al., 2006; Dose et al., 2009; Kumar et al., 2009; Andrade et al., 2013). Some of the limitations of our study are relatively smaller number of patients, timing of reassessment PET-CT was not uniform, and we did not study the dynamic contrast MRI which is an emerging tool. 

In conclusion 18F FDG PET-CT predicted the NACT response with greater accuracy than CT or clinical examination. It can be used effectively to identify non-PR early in the course so that toxicities of ineffective chemotherapy can be avoided, and other treatment options are explored.
